# Supplementary material for: Chromothripsis during telomere crisis is independent of NHEJ, and consistent with a replicative origin
Source: Genome Res. 2019 May;29(5):737–49. doi: 10.1101/gr.240705.118 (PMC6499312; doi:10.1101/gr.240705.118)
Supplement: Supplemental Material [file supp_gr.240705.118_Supplemental_file_1.zip › contigs/annotated_contigs/DB51/contig.2.DB51_length_284_mean_cov_4.78521126761.docx]

**DB51_length_284_mean_cov_4.78521126761**

```
AACAGAAAAAGGAACCACACATTTGTTTGTTTAATGAATGCCAAATGATAGACCTAGGTCTAACATAAAGCAGAGGAAATTATCTTTTC
 >chr18:66752215-66752372 - E=3e-83
TTGAGAGAGTATTGGTAATTGGCAATATCTGCCATGACTTGTTAGCATGTCCAACCTTCAATTCAGT|T|GATTGAGATGAAAATTATA
                                                                    >chr18:68495167-684
AGTAAGTGATTTTAAAAAATATGTTGTTAACTCAGTCCTCATCCTGTGCTTTTAAATTTCTTATCCAGGAAAATCACTTTTGACAGTGT
95295 - E=8e-66
GTCCTGACAAGGCATTTGC
```
